# A Novel Neurofilament Light Chain ELISA Validated in Patients with Alzheimer’s Disease, Frontotemporal Dementia, and Subjective Cognitive Decline, and the Evaluation of Candidate Proteins for Immunoassay Calibration

**DOI:** 10.3390/ijms23137221

**Published:** 2022-06-29

**Authors:** Shreyasee Das, Nele Dewit, Dirk Jacobs, Yolande A. L. Pijnenburg, Sjors G. J. G. In ‘t Veld, Salomé Coppens, Milena Quaglia, Christophe Hirtz, Charlotte E. Teunissen, Eugeen Vanmechelen

**Affiliations:** 1 ADx NeuroSciences NV, Technologiepark-Zwijnaarde 94, 9052 Gent, Belgium; shreyasee.das@adxneurosciences.com (S.D.); dewitnele@hotmail.com (N.D.); dirk.jacobs@adxneurosciences.com (D.J.); 2 Department of Clinical Chemistry, Neurochemistry Laboratory, Amsterdam University Medical Centres, Vrije Universiteit, Amsterdam Neuroscience Neurodegeneration, De Boelelaan 1117, 1081 HV Amsterdam, The Netherlands; yal.pijnenburg@amsterdamumc.nl (Y.A.L.P.); g.intveld1@amsterdamumc.nl (S.G.J.G.I.‘t.V.); c.teunissen@amsterdamumc.nl (C.E.T.); 3 National Measurement Laboratory, LGC Limited, Teddington TW11 0LY, UK; salome.coppens@lgcgroup.com (S.C.); milena.quaglia@lgcgroup.com (M.Q.); 4 IRMB-PPC, INM, CHU Montpellier, INSERM CNRS, 34295 Montpellier, France; c-hirtz@chu-montpellier.fr

**Keywords:** neurofilament-light, biomarker, immunoassay, ELISA, calibrator

## Abstract

Neurofilament light chain (Nf-L) is a well-known biomarker for axonal damage; however, the corresponding circulating Nf-L analyte in cerebrospinal fluid (CSF) is poorly characterized. We therefore isolated new monoclonal antibodies against synthetic peptides, and these monoclonals were characterized for their specificity on brain-specific intermediate filament proteins. Two highly specific antibodies, ADx206 and ADx209, were analytically validated for CSF applications according to well-established criteria. Interestingly, using three different sources of purified Nf-L proteins, a significant impact on interpolated concentrations was observed. With a lower limit of analytical sensitivity of 100 pg/mL using bovine Nf-L as the calibrator, we were able to quantify the Nf-L analyte in each sample, and these Nf-L concentrations were highly correlated to the Uman diagnostics assay (Spearman rho = 0.97, *p* < 0.001). In the clinical diagnostic groups, the new Nf-L ELISA could discriminate patients with Alzheimer’s disease (AD, *n* = 20) from those with frontotemporal lobe dementia (FTD, *n* = 20) and control samples with subjective cognitive decline (SCD, *n* = 20). Henceforth, this novel Nf-L ELISA with well-defined specificity and epitopes can be used to enhance our understanding of harmonizing the use of Nf-L as a clinically relevant marker for neurodegeneration in CSF.

## 1. Introduction

The neuronal cytoskeleton consists of neuronal intermediate filament proteins. There are several subunits of these intermediate filament proteins including low, medium, and high molecular weight neurofilaments, α-internexin, and peripherin [1]. Neurofilaments are present in the cytoplasm of neurons and are released into the interstitial fluid due to axonal damage [2]. The released neurofilaments travel to the cerebrospinal fluid (CSF) and, subsequently, in small amounts to the blood [3]. The exact pathological pathway of neurofilaments is yet to be deciphered, although, it has been discovered that defects in neurofilament subunit assembly and neurofilament light chain (Nf-L) gene mutations are major contributors to familial neurodegenerative disorders, such as Charcot–Marie–Tooth disease [4]. The Nf-L protein in particular is by far the most extensively evaluated neuronal intermediate filament protein in body fluids and has therefore emerged as a potent biomarker for several neurodegenerative diseases, as well as for the identification of clinical groups in comparison to normally aging brains [5,6,7]. While the turnover of Nf-L in normally aging brains is slow, abnormally elevated levels of Nf-L in CSF and blood have been associated with several neurodegenerative conditions such as multiple sclerosis (MS), various types of dementia such as Alzheimer’s disease (AD) and frontotemporal dementia (FTD), and several other neurodegenerative diseases [4,6,8]. Therefore, CSF, plasma, and serum bio-fluid samples are currently in use for the detection of Nf-L in diagnostic patient groups, as well as for disease monitoring [9,10,11,12,13].

Despite recent advancements in the quantification of serum and plasma Nf-L concentrations, the pathophysiological relevance of CSF remains unrivaled due to its proximity to the brain and the continuous exchange of proteins between the brain and CSF [14]. Furthermore, although there is a high correlation between the Nf-L levels in CSF and plasma, recent findings indicate that CSF Nf-L and plasma Nf-L associate differentially with biomarkers of neurodegeneration [15]. This revelation suggests that CSF Nf-L and plasma Nf-L may reflect distinct neuro-pathologies, emphasizing the need to understand the clinical implications of both fluid biomarkers.

Despite the elevated interest towards Nf-L as a potential biomarker for neurodegeneration, at present, there are only two antibody pairs recognized and validated for Nf-L immunoassays. The first is the commercial Nf-L antibodies from UmanDiagnostics that are being used in ELISA for CSF applications and on the Simoa platform (Quanterix) for ultra-sensitive quantification in plasma/serum [12,13,16]. The second is two monoclonal antibodies (mAb), NfL21 and NfL23, which are currently in use in a more recent ELISA for CSF measurements, although these antibodies are not yet available commercially [17]. In order to qualify any biomarker for use in a clinical cohort, it is of utmost importance to check the reproducibility of the Nf-L measurements on different assays and assay platforms [18].

The deep-proteome analysis of the tryptic digests of CSF proteins has revealed that C-terminal fragments of the core of the neurofilament rod are present in circulation [19]. These findings were corroborated in mouse models of neurodegeneration using immunoprecipitation and mass spectrometry techniques [11]. In addition, spiking experiments of the full-length neurofilament demonstrate that the full-length neurofilament is not stable in CSF [20]. For these reasons, we aimed to develop a mouse peptide-immunization method to generate new Nf-L specific antibodies. For immunization, we have used both short antigenic epitopes to design short synthetic peptides (19 amino acids) as well as long synthetic peptides (79 amino acids). The antibodies generated were screened for their reactivity to Nf-L not only against synthetic peptide sequences but also against purified bovine Nf-L (bNf-L). Our goal was to purify two complimentary mAbs to design a novel immunoassay for the quantification of Nf-L in CSF.

Herein, we describe the development and the analytical and clinical validation of an Nf-L ELISA using two novel antibodies purified in-house, ADx206 and ADx209. With this novel Nf-L ELISA that is highly sensitive and robust, we thus broaden the scope of available CSF Nf-L immunoassays. Preliminary experiments were carried out by mass spectrometry and western blot to confirm the sequence of the Nf-L from different manufacturers and to provide a preliminary indication of the relative protein content of the materials. The selection and characterization of the immunoassay calibrators are of paramount importance to ensure the robustness of the assay and long-term protein traceability. It also facilitates the development of potential reference measurement procedures [21]. To this end, as a first potential step towards harmonization, we evaluated the analytical performance of different sources of purified Nf-L proteins as calibrators in the immunoassay.

## 2. Results

### 2.1. ADx206 and ADx209 Antibodies Are Specific for Nf-L and Recognize Epitopes in the Coil 2/B Region of Nf-L

**Figure 1:**
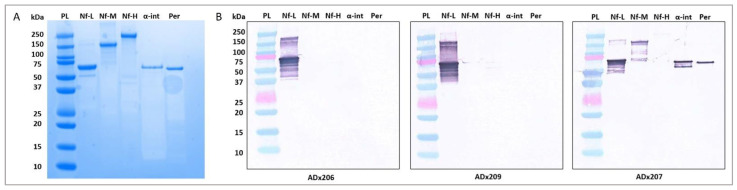
Specificity of ADx206 and ADx209. (**A**) SDS-PAGE of the native neurofilament proteins; 2 µg of protein was loaded in each lane. (**B**) Western blot evaluation of the reactivity of ADx206, ADx209, and ADx207 antibodies to native neurofilament proteins; 200 ng of each protein was loaded on the gel. Each blot contained the protein ladder (PL), Nf-L, Nf-M, Nf-H, α-internexin (α-int), and, finally, peripherin (Per). ADx206 and ADx209 are highly specific antibodies with no cross-reactivity to Nf-M, bNf-H, α-internexin, or peripherin. ADx207 antibody shows cross-reactivity with bNf-M, α-internexin, and the peripherin protein. The unloaded lanes in the gel and the blots have been cropped.

**Figure 2:**
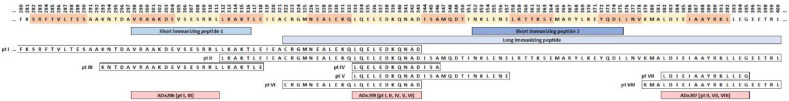
Epitope recognition sites of ADx206, ADx209, and ADx207 antibodies in the Coil 2/B region of Nf-L protein. The two short and one long peptide immunization sites (Section 4.1) are defined in the figure. Eight synthetic peptide sequences (pt I to pt VIII) defined in the figure were used to map the epitopes recognized by the antibodies.

We first checked the cross-reactivity of the generated Nf-L antibodies using western blot. The results shown in Figure 1 indicate that ADx206 and ADx209 are highly specific for native bNf-L binding. In contrast, for antibody ADx207, cross-reactivity was observed with other intermediate filament proteins such as bNf-M, α-internexin, and peripherin. The antibodies bind to epitopes in the coil 2/B sequence region of human Nf-L. ADx206 showed reactivity with epitopes of the biotinylated synthetic peptides (pt) pt I and pt III, while ADx209 reacted with pt II, IV, V, and VI and mildly with pt I (Figure 2, Table 1). As is clear from the peptide mapping, the epitopes recognized by the two antibodies are non-overlapping. This antibody pair covers a large detection range for Nf-L because ADx206 recognizes epitopes in the N-terminus of the coil 2/B region, while ADx209 recognizes epitopes towards the C-terminal of the coil 2/B region. The ADx207 antibody, on the other hand, shows reactivity with pt VII and VIII, which maps beyond the region of the neurofilament light chain in humans. These results corroborate the cross-reactivity of ADx207 that we have observed in the western blot. Therefore, we developed an ELISA using ADx206 as the capture and ADx209 as the detector antibody.

### 2.2. Analytical Performance Validation of the Nf-L ELISA

**Figure 3:**
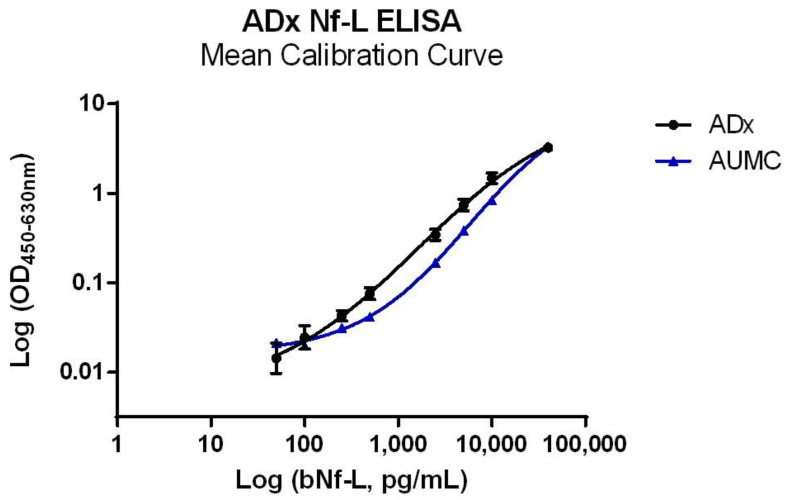
Mean calibration curve of full-length bNf-L in the ADx Nf-L ELISA determined at ADx and at AUMC. The calibrator curve has been fitted using a log-transformed, sigmoidal, unweighted 4PL curve fit. An analytical range of 40,000 pg/mL to 50 pg/mL was used.

**Figure 4:**
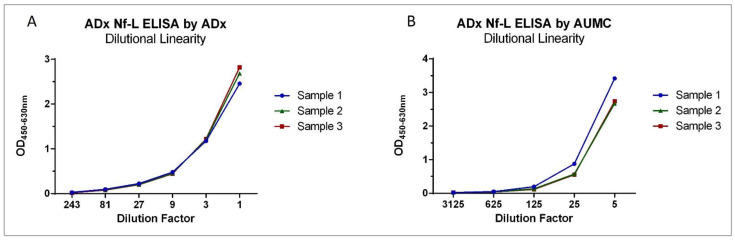
Dilutional Linearity. (**A**) Dilutional linearity of three CSF samples in the ADx Nf-L ELISA validated at ADx NeuroSciences. The three CSF samples were spiked with 30,000 pg/mL of bNf-L and maintained linearity within the acceptable range of 80–120% up to dilution factor 9, but at dilution factors of 21, 81, and 243, linearity deviated from the acceptable range (they were 137%, 127%, and 74% respectively). (**B**) Dilutional linearity of three CSF samples in the ADx Nf-L ELISA validated at AUMC. The samples were spiked with 100,000 pg/mL of bNf-L. All three samples maintained linearity within the acceptable range up to dilution factor 625, beyond which the signals dropped below their LLOQ.

**Figure 5:**
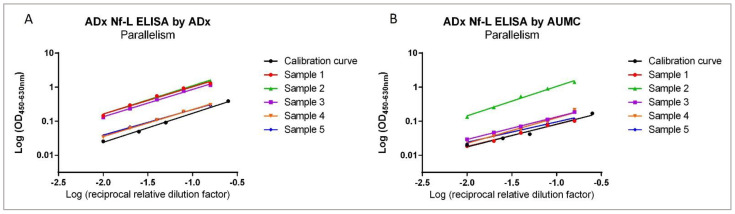
Parallelism. (**A**) Parallelism evaluated for five endogenous high CSF samples against the bNf-L calibrator at ADx NeuroSciences. For all five CSF samples, the percentage deviations were all within the predefined acceptable range, and, thus, the samples were parallel to the native bNf-L protein calibrator. (**B**) Parallelism evaluated for five endogenous high CSF samples against the bNf-L calibrator at AUMC. All CSF samples except one fell within the acceptable range of 80–120%.

At ADx as well as AUMC, the Nf-L ELISA was validated within an analytical range of 40,000 pg/mL to 50 pg/mL (Figure 3). Both sites used their available samples and validation criteria, and the calculations were performed as previously published [22]. Three different CSF samples were each spiked with three different bNf-L concentrations to evaluate the spike-recovery. The spike recovery was within the acceptance criteria for all the samples at both centers, except the low spike sample at ADx, where the mean recovery range was between 54 and 85%. The ELISA satisfied the precision criteria, as the mean variabilities for the intra-assay and inter-assay were below 15% at both validation centers. The mean dilutional linearity measured at ADx and AUMC was well within the acceptance criteria (80–120%) for all three CSF samples (Figure 4). The mean parallelism for the five CSF samples was determined to be 90% at ADx and 93% at AUMC, suggesting minimal matrix effects in the assay (Figure 5). The analytical assay validation results are detailed in Table 2.

### 2.3. ADx Nf-L ELISA Correlates with UmanDiagnostics Nf-L ELISA with a High Significance

**Figure 6:**
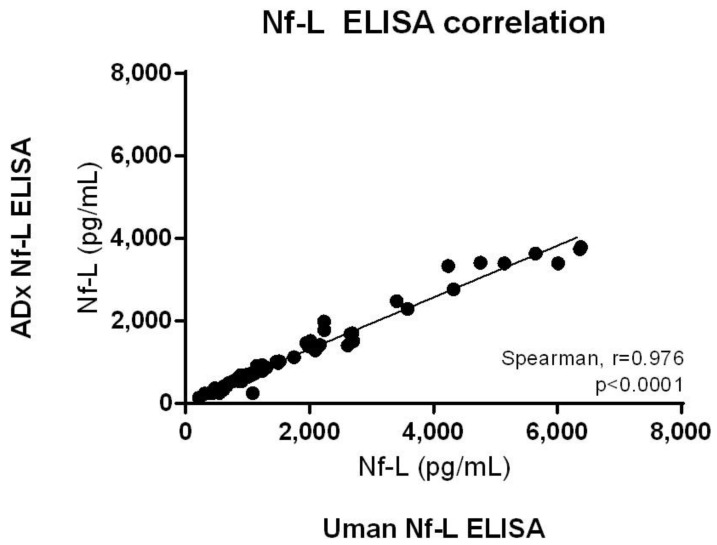
Correlation of 60 clinical samples measured with the ADx Nf-L ELISA and the commercial UmanDiagnostics Nf-L ELISA. The data were analyzed using the non-parametric Spearman correlation on GraphPad Prism v.6.07. The correlation is significant (Spearman rho = 0.976, *p* < 0.0001).

We measured 60 clinical CSF samples with the ADx Nf-L ELISA as well as the commercial UmanDiagnostics Nf-L ELISA kit. All clinical samples could be measured above the LLOQ of the ELISA. We found a strong and significant correlation between the ADx Nf-L ELISA and the UmanDiagnostics Nf-L ELISA (Spearman’s rho = 0.976; *p* < 0.0001) (Figure 6). There was no significant difference between the CSF Nf-L levels in the male versus female patient groups in this cohort, following a pairwise comparison and post hoc multiple comparisons (Table 3). Therefore, the variable ‘sex’ was not identified as a confounder.

### 2.4. Clinical Validation of the ADx Nf-L ELISA

**Figure 7:**
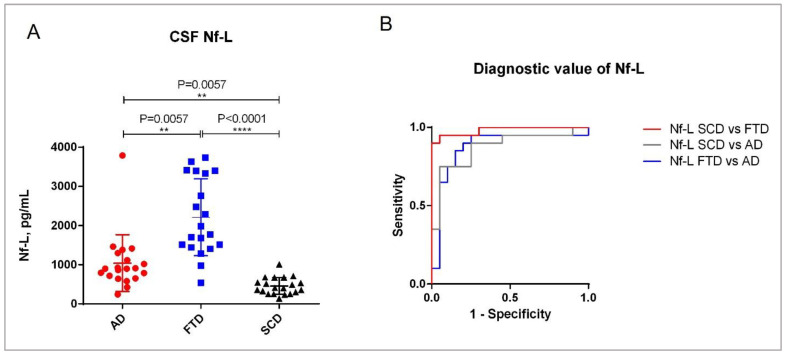
Clinical validation of the Nf-L ELISA. (**A**) CSF Nf-L concentrations in different diagnostic groups. The analysis was carried out using a Kruskal–Wallis analysis of variance (ANOVA) for multiple comparisons. (**B**) ROC curves. The graph shows the diagnostic value of CSF Nf-L in the clinical diagnostic groups AD and FTD, as well as in SCD. (On Graphpad Prism, **: *p* ≤ 0.01; ****: *p* ≤ 0.0001).

The demographics of the clinical cohort are described in Table 3. The post-hoc analysis in the cohort revealed that age differed between the patients with AD and SCD, with a significance of *p* = 0.03. As expected, the mini-mental state examination (MMSE) scores of patients were significantly different between SCD and FTD (*p* < 0.0001), as well as between SCD and AD (*p* < 0.0001). With the novel ELISA, we could differentiate between all three clinical diagnostic groups of AD, FTD, and SCD (Figure 7A). The difference between the clinical groups FTD and SCD was the most significant (*p* < 0.0001), while the significance of the difference between the groups AD versus FTD and AD versus SCD was equivalent (*p* = 0.0057). These results are also reflected by the diagnostic value of the biomarker CSF Nf-L amongst the clinical groups (Figure 7B, Table 4). Our analysis revealed that CSF Nf-L had a higher diagnostic significance for differentiating between the SCD and FTD groups (AUC = 0.98, *p* < 0.0001) than between the SCD and AD or FTD and AD groups (AUC = 0.88, *p* < 0.0001). Furthermore, the CSF Nf-L concentrations correlated moderately with the age of the patients (Spearman’s rho = 0.4, *p* = 0031) (Supplementary Information Figure S1A), indicating that Nf-L levels increase with the increase in the patient’s age. We also found a significant negative correlation between the CSF Nf-L concentrations and MMSE scores of the patients (Spearman’s rho = −0.50, *p* < 0.0001) (Supplementary Information Figure S1B).

### 2.5. ADx206 and ADx209 Antibodies React with All Three Calibrator Proteins

**Figure 8:**
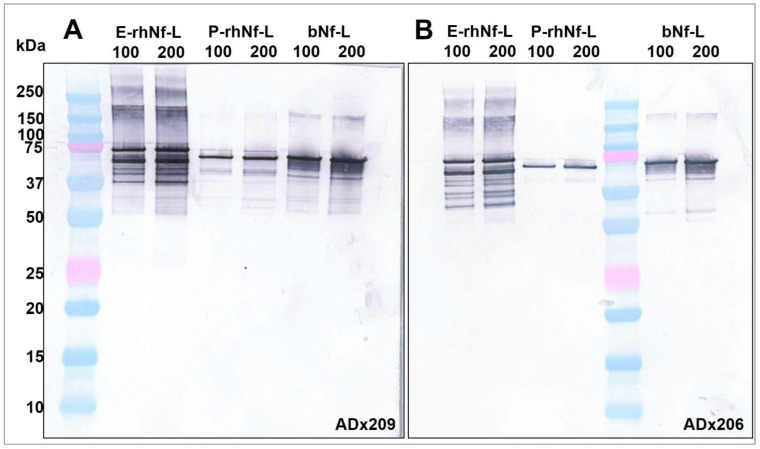
Western blot showing the reactivity of (**A**) ADx209 and (**B**) ADx206 antibodies to primary calibrators bNf-L (MyBioSource), E-rhNf-L (EnCor), and P-rhNf-L (Promise). For each calibrator, two lanes were loaded on the gel with 100 ng and 200 ng of protein, as indicated on the blot. Unloaded lanes have been cropped from the blots.

We characterized three abundant and commonly used purified Nf-L protein calibrators (Table 5). The calibrators were first checked for their reactivity with the Nf-L antibodies ADx206 and ADx209 by western blot (Figure 8). Both the antibodies recognized the epitopes of all three calibrators. In the western blot, we can see that E-rhNf-L and bNf-L show multiple bands that correspond to non-specific interactions, while P-rhNf-L does not. Given that the P-rhNf-L shows the least aspecificity, we may conclude that it contains a highly pure form of Nf-L. From Figure 8, the absolute Nf-L protein concentration of E-rhNf-L and bNf-L appears to be comparable, although these results are qualitative. The three preparations were tryptic digested and analyzed by liquid chromatography–mass spectrometry to confirm the sequence reported by the manufacturers. The coverage obtained is shown in Supplementary Information Figure S2, confirming that the main content of the calibrators is from Nf-L. By screening against a database, additional non-specific peptides were detected for the E-rhNf-L, indicating the lower purity of this preparation, which was also revealed by the western blot in Figure 8.

### 2.6. The Three Full-Length Nf-L Calibrators Show Differential Reactivity in ELISA

**Figure 9:**
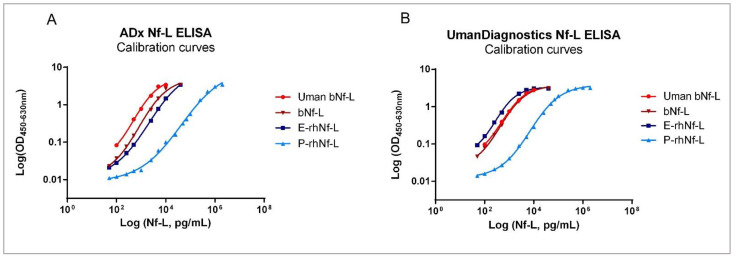
Evaluation of the primary calibrators by the ADx NeuroSciences Nf-L ELISA and the UmanDiagnostics Nf-L ELISA. Calibration curves of the three primary calibrators as well as the UmanDiagnostics standard bNf-L calibrator measured in (**A**) the ADx Nf-L ELISA and (**B**) the Undiagnostic Nf-L ELISA.

The three purified protein calibrators (Table 5) were measured with novel Nf-L ELISA as well as the UmanDiagnostics Nf-L ELISA kit. As an additional standard for comparison, the native bNf-L calibrator available in the UmanDiagnostics ELISA kit was also measured using our Nf-L ELISA. Using the P-rhNf-L protein as a calibrator, we found that it had a lower assay reactivity compared to the other Nf-L protein calibrators in both ADx as well as UmanDiagnostics Nf-L ELISAs. Consequently, the Nf-L concentration of the CSF samples interpolated by this calibration curve was significantly higher than that of those interpolated with the bNf-L, E-rhNf-L, or Uman bNf-L standards (Figure 9, Table 6). These findings were also reflected in the clinical diagnostic groups of AD, FTD, and SCD (Supplementary Information Figure S4).

## 3. Discussion

We have developed a novel and reliable Nf-L ELISA that can be used successfully for the measurement of clinical CSF samples. The Nf-L concentrations measured in CSF samples correlate well with those measured with the UmanDiagnostics Nf-L ELISA kit. The developed assay is sensitive and precise, and neither of the antibodies used in this assay show cross-reactivity with other neuronal intermediate filament proteins. Other validation parameters such as dilutional linearity, parallelism, and spike-recovery met the reference criteria set at both assessment centers, ADx and AUMC. Using this ELISA, we were able to successfully differentiate the clinical diagnostic groups of AD, FTD, and SCD. Our results corroborate previously reported findings that CSF Nf-L concentrations in FTD patients are much higher than those of people with AD [8,17,23]. In addition, as expected, AD and FTD patients had significantly higher CSF Nf-L concentrations than patients with subjective cognitive decline. Moreover, a recent mass-spectrometry-based characterization of CSF Nf-L revealed that the tryptic digested peptide sequence that correlated best with the UmanDiagnostics Nf-L ELISA was NfL324. This peptide lies within the peptide sequence covered by the ADx206 and ADx209 epitope sequences mapped in Figure 2. This further confirms the reasoning behind the strong correlation between the commercial ELISA and our novel Nf-L ELISA [24].

In our experimental setting, we have evaluated three primary protein calibrators for the quantification of CSF Nf-L. We found that there were significant differences among the calibrators while quantifying Nf-L in CSF samples in ELISA. The capture and detector antibodies ADx206 and ADx209 recognized the epitopes of all three of these calibrators, as was confirmed by western blot. In addition, the Nf-L protein calibrator sequences were confirmed by mass spectrometry, demonstrating the presence of the reactive epitopes in the preparations (Section 2.5).

Furthermore, we found that the reactivity of the P-rhNf-L calibrator was significantly lower, which resulted in elevated interpolated Nf-L concentrations, even though this recombinant Nf-L showed the least amount of aspecificity in the western blot. Thus, the concentrations determined by the P-rhNf-L calibrator were several times beyond the known clinical range of CSF Nf-L [3]. Given the self-assembling nature of neuronal intermediate filaments, one possibility concerning the reduced reactivity of P-rhNf-L could also be that the antibody binding epitopes of the protein calibrator were simply not exposed for binding to the Nf-L antibodies [1,25,26]. While the sequence of the calibrators was confirmed by liquid chromatography-mass spectrometry, further experiments are required to assess the effect of the presence of fragments/oligomers in the preparations used in immunoassays.

Considering that purified Nf-L proteins from bovine and human sources are predominantly used as standards in immunoassays, our preliminary findings raise important questions regarding the clinical implications of calibrator-specific differences [1,17,27,28]. The use of CSF Nf-L as a biomarker for neurodegeneration warrants the definition of clinical cut-off values for CSF Nf-L concentrations. To our knowledge, there is not yet an established method of Nf-L quantification where threshold CSF Nf-L concentrations are defined for diagnostic groups. In order to define these clinical CSF Nf-L ranges, however, we would need a harmonized Nf-L quantification method where the absolute Nf-L concentrations can be determined with high accuracy and traceability. Moreover, the European Union Regulation on In Vitro Diagnostic Medical Devices (Regulation[EU]2017/746) stipulates that a material used as a calibrator must be traceable to the higher-order reference material available [29].

Through our research, we have highlighted a novel and robust Nf-L ELISA that can be used to measure CSF Nf-L concentrations in several neurodegenerative diagnostic groups. The correlation between this novel assay and the commercially available assay is a good premise for the success of current harmonization and standardization initiatives that are critical requirements for the definition of reliable clinical thresholds. Our work also illustrates the source and supplier-specific differences in purified Nf-L proteins that exist in current immunoassays and may influence clinical results. An increase in CSF Nf-L levels in an individual over time is inherent to normal aging, and, in line with previous findings, we reported in this cohort that Nf-L levels increased with the increasing age of patients [13,30,31]. As a probable limitation, due to the small sample size, we were unable to correct for the age effect in this cohort. In addition, we did not see a significant correlation of CSF Nf-L with the cognitive (MMSE) scores of the patients within each diagnostic group. Although the sample set used was sufficient to validate the ELISA for clinical use, the diagnostic significance remains to be evaluated in follow-up studies. Further experiments are also necessary to determine if structural differences induced by chemical modifications or excipients in the preparation of the calibrators are responsible for the different immunoassay responses. A possible approach to this end may be to evaluate peptide sequences or pooled CSF lots as calibrators. Commutability studies across different immunoassays to quantify Nf-L in CSF as well as their correlation to Nf-L concentrations obtained by mass spectrometry would be an important step toward harmonizing the existing immunoassays in different platforms as well as defining clinically relevant Nf-L concentration ranges [14].

## 4. Materials and Methods

### 4.1. Generation of ADx206 and ADx209 Antibodies

All peptide immunizations in mice were carried out by Biotem (Apprieu, France) in compliance with the EU animal welfare legislation. The mice-bleeding and serum analysis experiments were performed in accordance with good animal practices, following strict guidelines and the approval of the ethical committee at Biotem. All research involving mice was performed in accordance with the ARRIVE guidelines [32].

In order to generate de-novo Nf-L mAbs, we applied two peptide immunization approaches to female OF1 mice. One is based on short (19 amino acids) antigenic peptides. The second approach involves long peptide (79 amino acids) immunization and is based on the previous experience that sometimes longer peptides generate antibodies that are more likely to be reactive with the native antigen. While the sequences of the peptides are based on the neurofilament sequence, the N- and C-terminus of the peptides are often highly immunogenic and not present in the natural protein. Therefore, during the titration and screening for candidate antibodies, we used plates coated with purified bNf-L to select mice for fusion and candidate hybridomas for cloning. All peptides selected for immunization started with a cysteine residue which allows for the coupling to a Keyhole limpet hemocyanin (KLH) carrier protein to improve the immune response of peptide antigens. In two short antigenic peptides (V298-T316; 19AA and N352-L375; 24AA) and two of the longer peptides (I213-A297; 85AA and L258-D343; 86AA), an additional cysteine was added for conjugation with the KLH carrier protein. For the third long peptide (C322-L400; 79AA), the natural cysteine residue at position 322 was used. Five mice were immunized for each peptide sequence. The serum of each mouse was titrated against the respective peptide counterparts and against purified bNf-L (MyBioSource, San Diego, CA, USA). For each immunization strategy, two mice with the highest titers against both peptide and bNf-L were boosted after several weeks and were then sacrificed for classical hybridoma fusion. The resulting hybridoma clones were screened for their reactivity against bNf-L and their peptide, and, per strategy, 5 to 10 candidate clones were selected and stored. Of these selected clones, two were subcloned to produce monoclonal antibodies which were further characterized. Their reactivity against brain extracts and their specificity for Nf-L were tested using western blotting. The check for selectivity on these other brain-specific intermediate filaments was needed because the selected region of the immunogenic peptide contains an epitope cross-reactive with the other intermediate filaments, and there is evidence that neurofilament medium (Nf-M) and neurofilament heavy (Nf-H) chain proteins are also circulating in CSF, perhaps as heteropolymers of different intermediate filaments [33]. A final antibody selection was made by pairing the candidates in a sandwich ELISA in multiple screenings and choosing the combination with the highest reactivity on both calibrator and routine CSF samples, resulting in the combination of ADx206 as the capture and ADx209 as the detector antibody.

### 4.2. Western Blotting

Western blotting was used to check for the cross-reactivity of the two Nf-L antibodies ADx206 and ADx209 with the major neuronal intermediate filament proteins such as Nf-M, Nf-H, α-internexin, and peripherin, as well as to compare the three calibrator proteins. For the intermediate filament proteins, we also used ADx207 as a cross-reactive positive control antibody. Native bNf-L, bNf-M, and bNf-H (MyBioSource, San Diego, CA, USA), α-internexin (EnCor Biotechnology, Gainesville, FL, USA), peripherin (EnCor Biotechnology, Gainesville, FL, USA), E-rhNf-L, and P-rhNf-L were heated for 5 min at 96 °C in a sample loading buffer (Cell Signalling Technologies, Leiden, The Netherlands) under reducing conditions; next, they were loaded in Bolt 10% Bis-Tris plus Gels (Invitrogen, Life Technologies, Merelbeke, Belgium) and run at 120 volts for 45 min. The immobilized proteins on the gel were then transferred by electrophoretic transfer to a Transfer Pack membrane (Bio-Rad, Temse, Belgium, 1704159) using the Trans-Blot^®^ Turbo™ system (Bio-Rad) for 7 min. The blots were blocked for 30 min with 5% skimmed milk in DPBS and incubated with a 1 µg/mL solution of the primary antibodies ADx206, ADx207, or ADx209 for 1.5 h. Goat anti-mouse-Alkaline Phosphatase labeled antibody (Jackson Immunoresearch, Suffolk, UK, 115-055-003) was used as the secondary antibody conjugate, and the blots were incubated in a 1:5000 dilution of this antibody for 1 h. Finally, 1 mL of the NBT-BCIP chromogenic solution (Thermofisher Scientific, Life Technologies, Merelbeke, Belgium, 34042) was added to the blots and was allowed to develop for 10 min.

### 4.3. Nf-L Sandwich ELISA and Indirect ELISA for Epitope Mapping

We developed an in-house sandwich ELISA for the measurement of Nf-L in CSF samples. In this assay, ADx206 was used as a capture antibody and ADx209 was used as the detector antibody, which was biotinylated with Sulfo-NHS-LC-LC-Biotin at 120× molar excess (Thermofisher Scientific, Life Technologies, Merelbeke, Belgium, 34042, A35358) following a protocol previously described [34]. A total of 100 µL of capture antibody, diluted in Dulbecco’s phosphate-buffered saline (DPBS) to 5 µg/mL (Lonza, Verviers, Belgium, BE17-512F), was coated overnight at 4 °C on NUNC maxisorp F8 strips. After coating, each well was washed three times with 300 µL of the wash buffer composed of DPBS and 0.05% Tween20 (Merck Millipore, Overijse, Belgium, 8.22184.0500), tapped completely dry, and blocked for 2 h at room temperature (RT) with 300 µL of blocking buffer composed of 0.5% Blocker™ Casein in DPBS (Thermofisher Scientific, 37528) and 0.05% ProClin™300 (Sigma Aldrich, Merck Life Science BV, Overijse, Belgium, 48914-U). After blocking, the plate was tapped dry, and the calibrator series and CSF samples were added to the plate in a 1:1 dilution with a sample diluent composed of 1XDPBS, 0.1% Blocker™ Casein, 0.1% Tween20, and 0.05% ProClin™300. All samples and calibrators were measured in duplicate. The samples and calibrators were incubated for 1 h at RT and shaken at 800 rpm (Quanterix Digital Incubator Shaker, Lexington, MA, USA, 230V), followed by a wash step, as described earlier. A total of 100 µL of biotinylated ADx209 detector antibody was added to each well at a final concentration of 200 ng/mL, diluted in the sample diluent. The plate was then incubated for 45 min at RT and shaken at 800 rpm. After another wash, 100 µL of 50 ng/mL Streptavidin Poly-Peroxidase conjugate (Stereospecific Detection Technologies Reagents, Baesweiler, Germany, SP80C) was added to each well, diluted in conjugate diluent (1XDPBS, 0.1% Blocker™ Casein, 0.05% ProClin™300), and incubated for 30 min under similar shaking conditions. Following a final wash step, 100 µL of chromogenic substrate 3,3′,5,5′-Tetramethylbenzidine (TMB, Stereospecific Detection Technologies Reagents, eEsTMB) was added to each well and shaken at RT for 15 min. The reaction was stopped by adding 50 µL of 1 M H_2_SO_4_. The OD values were measured at a wavelength of 450 nm against a reference wavelength of 630 nm using the CLARIOstar plate reader (BMG Labtech, Ortenberg, Germany).

In order to map the linear epitope recognition sites of ADx206 and ADx209, synthetic peptides following sequences in the coil 2/B region of Nf-L were custom synthesized at Proteogenix SAS. These N-terminal biotinylated peptides were delivered in lyophilized form, reconstituted in 50% DMSO, and stored at −20 °C. An indirect ELISA was carried out to map the epitope recognition sites for the ADx206 and ADx209 antibodies. F96 ELISA plates were coated overnight with 5 µg/mL of Streptavidin (Roche, Vilvoorde, Belgium, 1 mg/mL). After the plate was blocked, 20 ng/mL of the biotinylated peptides were added and incubated for 1 h at room temperature. Thereafter, the plate was washed, and 100 ng/mL of the Nf-L antibodies ADx206, ADx209, and ADx207 were added. The plate was incubated for another hour at room temperature. After another wash step, the reaction was detected with a 5000× diluted secondary conjugate goat anti-mouse Fc specific IgG antibody (Jackson Immunoresearch, Suffolk, UK, 115-035-071) incubated for 1 h in the same manner. The plate was washed one final time, 100 µL of TMB was added as the chromogenic substrate, and the plates were incubated for 30 min in the dark. The reaction was stopped, and the OD values were measured as described earlier.

Three purified Nf-L proteins were evaluated as calibrators for this Nf-L ELISA Immunoassay (Table 5). These calibrator proteins were serially diluted in the aforementioned sample diluent. The nominal concentration of P-rhNf-L was defined as 100 µg/mL.

### 4.4. Analytical Validation of the Nf-L ELISA

The novel Nf-L ELISA was analytically validated at ADx and AUMC following the methods discussed previously by Andreasson et al. [22]. The validation included the following aspects: precision (intra- and inter-assay variability), assay sensitivity and determination of the LLOQ, dilutional linearity, parallelism, and spike-recovery. In accordance with the internal quality control standards at ADx and AUMC, the acceptable range of variation in precision, %CV, was set at ≤15%. Similarly, the acceptable range of deviation for dilutional linearity, parallelism, and spike-recovery was set at 80% to 120%. OD values that were below the LLOQ of the ELISA were not taken into consideration while analyzing the validation criteria. In order to check for spike-recovery, three different CSF samples were spiked with low, medium, and high concentrations of stock bNf-L. The ELISA precision was checked by determining the intra-assay and inter-assay variability using three CSF samples covering the calibrator range. To determine the dilutional linearity, three endogenous low samples were spiked with a high concentration of bNf-L and diluted until the concentration was below the LLOQ. To evaluate the assay for matrix effects, a parallelism experiment was carried out with five endogenous high samples, which were diluted parallel with the calibrator, and the slopes were compared. The ADx Nf-L ELISA was further validated by the quantification of clinical CSF samples and by measuring its correlation with the commercial UmanDiagnostics Nf-L ELISA [35,36].

### 4.5. Selection of CSF Samples and Clinical Study Design

For the analytical validation of the Nf-L ELISA and the evaluation of the primary calibrators, routine CSF samples (Biomnis) available at the biobank of ADx were used. For these routine CSF samples, only age and gender data were available, with no additional clinical information or cognitive scoring. For clinical validation, a cohort of 60 CSF samples was used. This cohort contained three diagnostics groups (FTD, AD, and patients with subjective cognitive decline (SCD)) with every 20 samples in each group. The AD biomarker profile data (tau and amyloid-beta levels) and the paired MMSE scores (ranging from 6 to 30) were available for these patient samples. The clinical diagnosis of the samples was available from the AUMC biobank according to previously established diagnostic criteria for AD, FTD, and SCD [37,38,39]. The core diagnostic biomarker profile, as defined by AUMC, was based on the CSF Amyloid-beta 42, Tau, and pTau concentrations of the patients. The demographic characteristics of the clinical samples used are tabulated in Table 6. The clinical sample analysis was conducted blind for the diagnosis, and the samples were randomized.

## Figures and Tables

**Table 1:** OD450 nm–650 nm values obtained from the indirect ELISA for epitope mapping.

Antibody	OD							
	pt I	pt II	pt III	pt IV	pt V	pt VI	pt VII	pt VIII
ADx206	3.387	0.016	3.455	0.015	0.014	0.016	0.014	0.014
ADx207	0.016	0.211	0.018	0.016	0.013	0.015	2.146	2.500
ADx209	0.961	3.313	0.015	3.269	3.462	3.323	0.013	0.018

The reactivity of the ADx206, ADx209, and ADx207 antibodies with the synthetic biotinylated peptides (pt) are shown as a color gradient. ADx206 reacts with pt I and pt III. ADx209 reacts with pt II, IV, V, and VI and mildly with pt I. ADx207 reacts with pt VII, and VIII and mildly with pt II, which map beyond the region of the neurofilament light chain.

**Table 2:** Results Validation ADx Nf-L ELISA.

Parameter	Acceptance Criteria	ADx			AUMC		
**Sensitivity—LLOQ Conc**	Mean_blank_ + 10 × SD_blank_	148			348		
**Spike-Recovery** Low spike Medium spike High spike	80–120% recovery	**Conc (recovery %)**			**Conc (recovery %)**		
		283.1 (54–85%)			665 (100–131%)		
		867.8 (61–96%)			3508 (100–106%)		
		5231.2 (76–98%)			15,710 (94–99%)		
**Precision** Low sample Medium sample High sample	CV < 20%	**Conc.**	**Intra-assay**	**Inter-assay**	**Conc.**	**Intra-assay**	**Inter-assay**
		165	10.6%	15.4%	450	24.9%	24.9%
		487	4.3%	4.3%	2514	3.9%	8.3%
		1316	3.8%	3.8%	14,420	2.9%	10.7%
		Mean	6.2%	7.8%	Mean	10.5%	14.6%
**Dilutional Linearity**	80–120% linearity	**Mean 3 samples**			**Mean 3 samples**		
		**DF (x)**		**%L**	**DF (x)**		**%L**
		1		-	1		-
		3		105	5		-
		9		106	25		118
		27		137	125		114
		81		127	625		97
		243		74	3125		106
		Mean		110%	Mean		109%
**Parallelism** R^2^ Slope Range	80–120% in range with the slope of the calibrator	**Mean 5 samples**			**Mean 5 samples**		
		**Mean**		**SD**	**Mean**		**SD**
		0.993		0.006	0.986		0.012
		0.795		0.030	0.703		0.105
		90%		3%	93%		14%

All concentration values are in pg/mL. The table shows the LLOQ of the ELISA, the mean spike-recovery for three different CSF samples with low, medium, and high Nf-L protein spikes, the precision as the mean %CV of three CSF samples (intra-assay and inter-assay), the mean dilutional linearity for three CSF samples against their dilution factors, and the mean %parallelism with the calibrator for five CSF samples (SD = Standard Deviation, R^2^ = linear regression coefficient).

**Table 3:** Demographic characteristics of the clinical cohort.

		Diagnostic Groups					
Demographic Characteristics	AD	FTD	SCD	ANOVA *p*-Value	*p*-Value SCD vs. FTD	*p*-Value SCD vs. AD	*p*-Value AD vs. FTD
***n***	20	20	20	-	-	-	-
**Age**	66 (58, 75)	64 (61, 68)	55 (51, 66)	0.02	ns	0.0294	ns
**Sex, female**	10 (50%)	10 (50%)	5 (25%)	ns	ns	ns	ns
**MMSE**	21 (11, 25)	21 (17, 26)	29 (27, 30)	<0.0001	<0.0001	<0.0001	ns
**Amyloid-beta 42**	494 (443, 604)	846 (723, 1087)	1036 (870, 1101)	<0.0001	ns	<0.0001	<0.0001
**Tau**	613 (516, 980)	385 (265, 482)	190 (159, 234)	<0.0001	0.0031	<0.0001	0.0146
**pTau**	83 (61, 111)	45 (38, 66)	35 (26, 42)	<0.0001	0.0136	<0.0001	0.0064
**Nf-L**	899 (702, 1160)	1880 (1495, 3346)	409 (292, 578)	<0.0001	<0.0001	0.0057	0.0057

The data for the sample size are presented as *n* (%) or as the median (interquartile range). Age is shown in years and the fluid biomarker values are shown as pg/mL. *p*-Values were calculated using Kruskal–Wallis’ one way ANOVA and Dunn’s multiple comparison. (ns: not significant).

**Table 4:** AUC and *p*-values of ROC curves comparing the diagnostic value of CSF Nf-L between clinical groups.

Biomarker	Diagnostic Groups	AUC	*p*-Value
Nf-L	SCD vs. FTD	0.98	*p* < 0.0001
Nf-L	FTD vs. AD	0.88	*p* < 0.0001
Nf-L	SCD vs. AD	0.88	*p* < 0.0001

AUC values were calculated from ROC curves plotted in GraphPad Prism v.6.07 (AUC = Area under ROC curve).

**Table 5:** Full-length neurofilament light chain calibrators evaluated for Nf-L ELISA.

Protein	Supplier	Catalog No.	Characteristics	Cal. Range	Abbreviation
Bovine Nf-L	MyBioSource	MBS537339	Native Nf-L isolated from bovine spinal cord	40,000–50	bNf-L
Recombinant human Nf-L	EnCor Biotechnology	Prot-r-Nf-L	Recombinant Nf-L, His tagged, purified from *E. coli*	40,000–50	E-rhNf-L
Recombinant human Nf-L	Promise Proteomics	NF169530	Recombinant Nf-L, His tagged, purified from *E. coli*, received from LGC	2,000,000–50	P-rhNf-L

The calibrator range is shown in pg/mL.

**Table 6:** Comparison of primary calibrators by ADx Nf-L ELISA and UmanDiagnostics Nf-L ELISA in 14 unclassified CSF samples.

Dunn’s Multiple Comparisons Test	ADx Nf-L ELISA		UmanDiagnostics Nf-L ELISA	
	Significance	*p*-Value	Significance	*p*-Value
Uman bNf-L vs. P-rhNf-L	***	0.0002	****	<0.0001
Uman bNf-L vs. bNf-L	ns	>0.9999	ns	>0.9999
Uman bNf-L vs. E-rhNF-L	ns	>0.9999	ns	0.4188
P-rhNf-L vs. bNf-L	**	0.001	**	0.0012
P-rhNf-L vs. E-rhNf-L	****	<0.0001	*	0.0179
bNf-L vs. E-rhNf-L	ns	>0.9999	ns	>0.9999

Analysis was performed on GraphPad Prism v.6.07 using Kruskal–Wallis’ one way ANOVA and Dunn’s multiple comparisons test for non-parametric datasets. The greater the number of stars (*), higher the significance (On GraphPad Prism, *: *p* ≤ 0.05; **: *p* ≤ 0.01; ***: *p* ≤ 0.001; ****: *p* ≤ 0.0001); ns: not significant).

## Data Availability

The datasets generated during and/or analyzed during the current study are available from the corresponding author on reasonable request.

## Supplementary Materials

The following supporting information can be downloaded at: https://www.mdpi.com/article/10.3390/ijms23137221/s1.

## Abbreviations

ADx NeuroSciences (ADx), Alzheimer’s disease (AD), amino acids (AA), Amsterdam University Medical Centre at Amsterdam, The Netherlands (AUMC), amyotrophic lateral sclerosis (ALS), bovine neurofilament light (bNf-L), cerebrospinal fluid (CSF), subjective cognitive decline (SCD), frontotemporal dementia (FTD), keyhole limpet hemocyanin (KLH), monoclonal antibody (mAb), mini-mental state examination (MMSE), multiple sclerosis (MS), neurofilament heavy (Nf-H), neurofilament light (Nf-L), neurofilament medium (Nf-M), phosphorylated tau (pTau), recombinant human Nf-L from EnCor Biotechnology (E-rhNf-L), recombinant human Nf-L from Promise Proteomics (P-rhNf-L).
