# Supplementary material for: A Novel Neurofilament Light Chain ELISA Validated in Patients with Alzheimer’s Disease, Frontotemporal Dementia, and Subjective Cognitive Decline, and the Evaluation of Candidate Proteins for Immunoassay Calibration
Source: Int J Mol Sci. 2022 Jun 29;23(13):7221. doi: 10.3390/ijms23137221 (PMC9266977; doi:10.3390/ijms23137221)
Supplement: Supplementary file 1 [file ijms-23-07221-s001.zip › ijms-1737048-supplementary.pdf]

International Journal of
Molecular Sciences

MDPI

Article

A Novel Neurofilament Light Chain ELISA Validated in Patients with Alzheimer's Disease, Frontotemporal Dementia, and Subjective Cognitive Decline, and the Evaluation of Candidate Proteins for Immunoassay Calibration

Shreyasee Das [1], Nele Dewit [1], Dirk Jacobs [1], Yolande A. L. Pijnenburg [2], Sjors G. J. G. In 't Veld [2], Salomé Coppens [3], Milena Quaglia [3], Christophe Hirtz [4], Charlotte E. Teunissen [2] and Eugeen Vanmechelen [1,*]

[1] ADx NeuroSciences NV, Technologiepark-Zwijnaarde 94, 9052 Gent, Belgium; shrey-asee.das@adxneurosciences.com (S.D.); dewitnele@hotmail.com (N.D.); dirk.jacobs@adxneurosciences.com (D.J.); eugeen.vanmechelen@adxneurosciences.com (E.V.M.)

[2] Department of Clinical Chemistry, Neurochemistry Laboratory, Amsterdam University Medical Centres, Vrije Universiteit, Amsterdam Neuroscience Neurodegeneration, De Boelelaan 1117, 1081 HV Amsterdam, The Netherlands; yal.pijnenburg@amsterdamumc.nl (Y.A.L.P.); g.intveld1@amsterdamumc.nl (S.G.J.G.I.'t.V.); c.teunissen@amsterdamumc.nl (C.E.T.)

[3] LGC Limited, National Measurement Laboratory , Teddington TW11 0LY, UK; sa-lome.coppens@lgcgroup.com (S.C.); milena.quaglia@lgcgroup.com (M.Q.)

[4] IRMB -PPC, INM, Univ Montpellier, CHU Montpellier, INSERM CNRS, 34295 Montpel-lier , France; c-hirtz@chu-montpellier.fr

* Correspondence: eugeen.vanmechelen@adxneurosciences.com; Tel.: +32-9-2616988

Citation: Das, S.; Dewit, N.; Jacobs, D.; Yolande A.L. Pijnenburg; Sjors G. J. G. In 't Veld; Coppens, S.; Quaglia, M.; Hirtz, C.; Teunissen, C.E.; Vanmechelen, E. A Novel Neurofilament Light Chain ELISA Validated in Patients with Alzheimer's Disease, Frontotemporal Dementia, and Subjective Cognitive Decline, and the Evaluation of Candidate Proteins for Immunoassay Calibration. *Int. J. Mol. Sci.* **2022**, *23*, 7221. https://doi.org/10.3390/ijms23137221

Academic Editor: Thomas Fath

Received: 6 May 2022
Accepted: 27 June 2022
Published: 29 June 2022

Publisher's Note: MDPI stays neutral with regard to jurisdictional claims in published maps and institutional affiliations.

Copyright: © 2022 by the authors. Licensee MDPI, Basel, Switzerland. This article is an open access article distributed under the terms and conditions of the Creative Commons Attribution (CC BY) license (https://creativecommons.org/licenses/by/4.0/).

SUPPLEMENTARY INFORMATION

Materials and Methods

Materials used for Mass spectrometry (MS) analysis of Nf-L Calibrator proteins: Three Nf-L preparations and one isotopically labeled Nf-L were used for this purpose. These proteins are as follows: Human Neurofilament light polypeptide unlabelled and labeled on lysine and arginine with 13C and 15N (Promise Proteomics, Grenoble, FR)), Full-length recombinant protein Neurofilament Nf-L (EnCor Biotechnology Inc., Gainesville, FL, USA) and 68kDa Neurofilament Ag Bovine (MyBioSource Inc., San Diego, CA, USA). For proteolytic digestion, dithiothreitol (DTT) and iodoacetamide (IAA) were purchased from Sigma-Aldrich (Saint-Louis, MO, USA), Rapi Gest from Waters (Milford, MA, USA), and Trypsin Gold from Promega (Madison, WI, USA).

Characterization of Nf-L standard solutions by proteolytic digestion and liquid chromatography MS analysis: To verify the sequence of the purchased proteins, a nominal amount of 2µg of protein was first reduced with 2.5mM DTT and denatured using Rapi Gest (20:1 ratio reagent: protein) and equilibrated for 30 minutes at 60°C. The protein was then alkylated with 5.5mM Iodoacetamide (IAA) for 45 minutes in the dark and digested overnight by adding Trypsin Gold (Promega) (1:1 protein: trypsin). 10 µL of digested Nf-L were injected into a Vanquish Ultra-High-Performance Liquid Chromatography (UHPLC) system (Thermo Fischer Scientific) on an Acquity UHPLC C18 BEH 2.1x150mm column (Waters) coupled to an Orbitrap Q Exactive Plus (Thermo Fischer Scientific). The mobile phases were composed of water and acetonitrile with 0.1% formic acid.

The chromatographic gradient was 3 %B to 50 %B in 30 minutes and flowrate 400uL/min. Peptides were ionized by heated electrospray ionization (HESI) positive using the following parameters: Sheath gas flow rate: 25; Aux gas flow rate: 10; capillary temperature 310°C; spray voltage; 2.5kV. Spectra were acquired in Data Dependent Acquisition (DDA) with a mass range between 200 and 1800 m/z and with a 2 m/z quadrupole isolation mode. Data analysis was carried out through PEAKS Studio 7.5 (Bioinformatics Solutions) by using the human Nf-L sequence (Uniprot accession number: P07196). Differences between the human and the bovine Nf-L were taken into account.

Results

Supplementary Information Figure S1

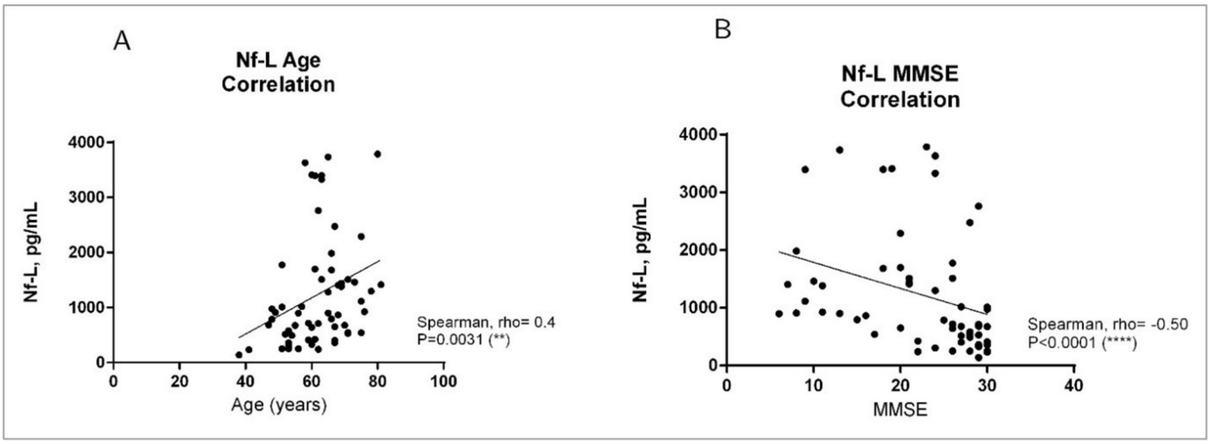

Supplementary Information Figure S1: Correlation of CSF Nf-L with Age and MMSE scores of patients. A)There is a significant correlation between the age of patients and their CSF Nf-L levels. B)There is a significant negative correlation between the Nf-L concentration and MMSE scores of patients, implying that Nf-L level rises with cognitive decline (lower MMSE score). (On Graphpad Prism, * : P ⩽ 0.05; ** : P ⩽ 0.01; *** : P ⩽ 0.001; **** : P ⩽ 0.0001.)

Supplementary Information Figure S2 shows the peptide coverage obtained for the three Nf-L calibrator protein preparations. The Nf-L standard sequence was confirmed by proteolytic digestion and analysis via LC-MS/MS. Sequence coverage was found to be 79% for the P-rhNf-L, 69% for the bNf-L, and 64% for the E-rhNf-L. We also screened the obtained sequences against Bos taurus (UP000009136) and Escherichia coli (E. coli, UP000033068) proteome databases to check for the presence of potential high-level impurities, although none could be identified in the preparations. Peptides from an E. coli protein were identified in the E-rhNf-L preparation with a sequence coverage of 60%.

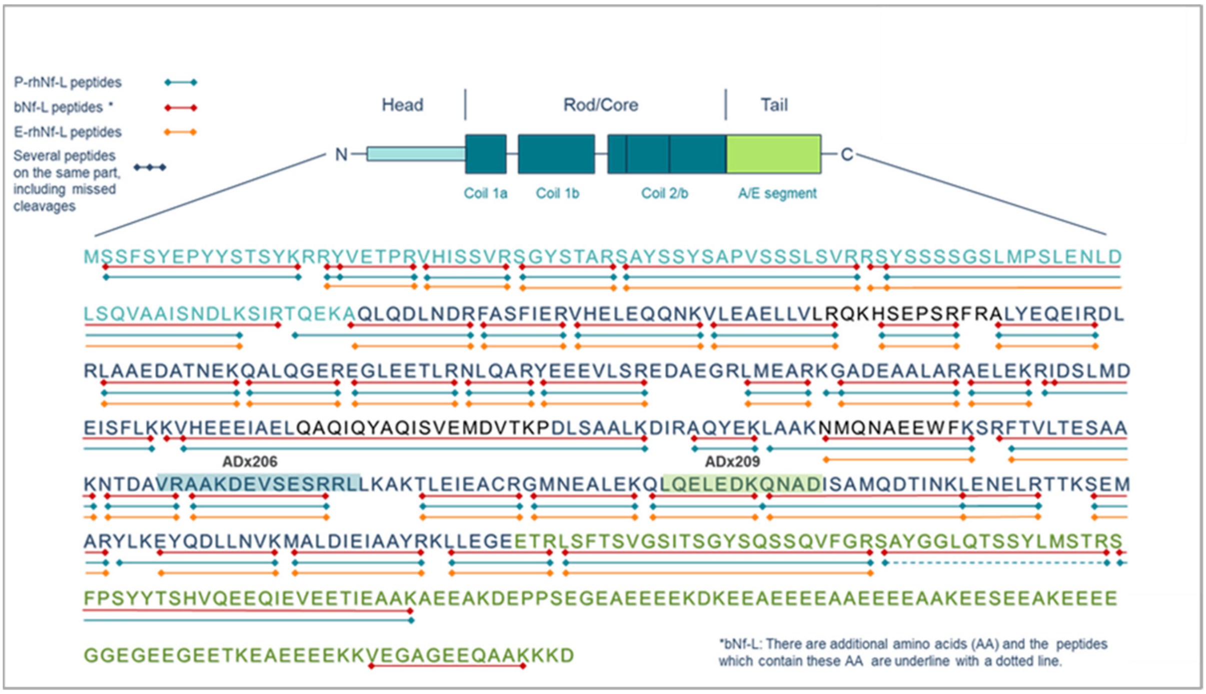

Supplementary Information Figure S2: Peptide mapping of the P-rhNf-L, E-rhNf-L, and bNF-L. A peptide map of the calibrator proteins was obtained following tryptic digestion of the three Nf-L proteins overnight and injection of the peptides into a UHPLC Vanquish system on a C18 column coupled to an Orbitrap Q-Exactive Plus. The identified peptides are mapped against the human Nf-L sequence (Uniprot entry: P07196). The identified peptides of the P-rhNf-L are underlined in red, the E-rhNf-L are in yellow and the bNf-L are in green. One bNf-L peptide is underlined with a dotted line as the bovine sequence contains additional amino acids on this part of the sequence. The epitopes of antibodies used in the immunoassay ADx206 and ADx209 are highlighted in blue and green respectively.

Supplementary Information Figure S3 shows that both Nf-L calibrators were tryptic digested in presence of the isotopically labeled Nf-L and monitored in DDA. The ratios of the area of the extracted ion peak chromatograms of 12 selected Nf-L peptides for the natural and labeled peptides are shown for the P-rhNf-L and the bNf-L material in the figure below. The higher the ratio of natural/labeled Nf-L, the higher is the expected concentration of the Nf-L protein in the sample. From these results, it appears that the Nf-L protein content is consistently higher for P-rhNf-L than for bNf-L. This analysis could not be performed for E-rhNf-L due to the presence of interfering peptides from E.coli already detected after a search against a database.

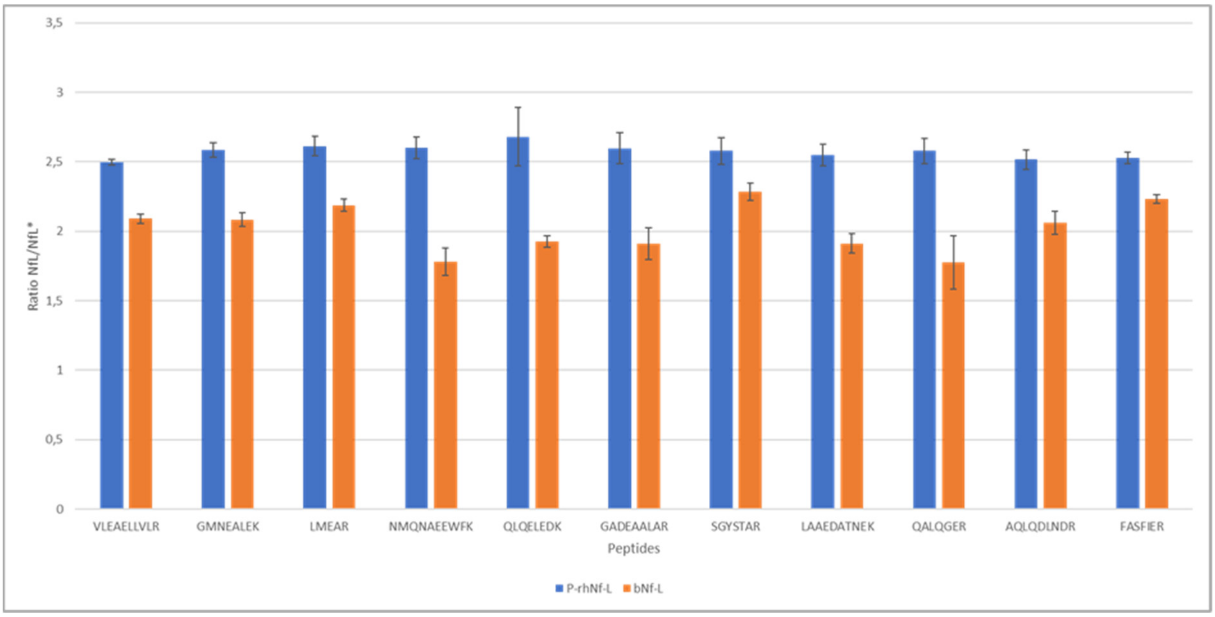

Supplementary Information Figure S3: Tryptic digestion of bNf-L and P-rhNf-L in the presence of isotopically labeled Nf-L. The figure shows the ratio of natural/labeled extracted peak chromatograms areas of peptides from the P-rhNf-L (blue) and bNf-L material (orange) injected in triplicate. Error bars represent the standard deviation between the value natural/labeled ratio of each injection.

Supplementary Information Figure S4

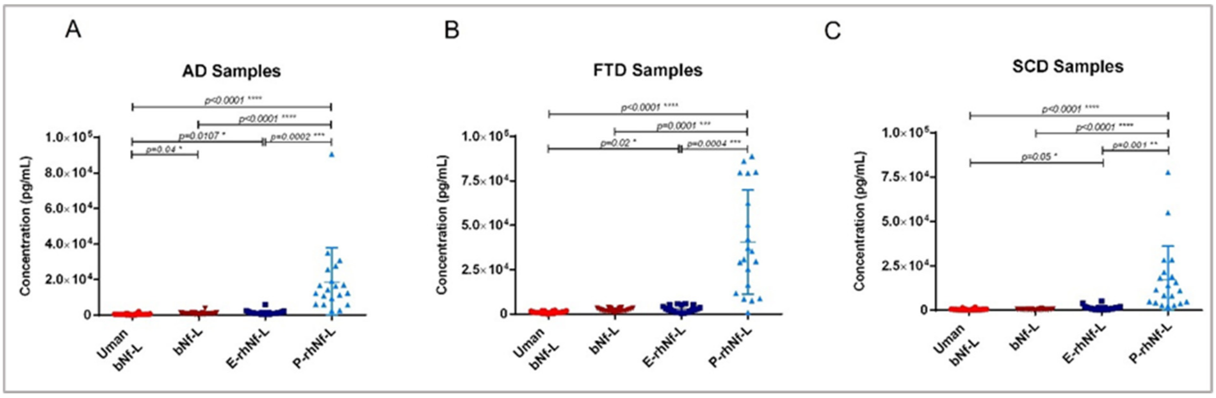

Supplementary Information Figure S4: Calibrator-specific differences in the quantification of CSF Nf-L concentrations measured with the ADx Nf-L ELISA. A) AD samples, B) FTD samples and C) SCD samples. Similar results with the UmanDiagnostic Nf-L ELISA are not shown. The group comparisons were performed using Kruskal-Wallis' ANOVA. (On Graphpad Prism, * : $P \leqslant 0.05$; ** : $P \leqslant 0.01$; *** : $P \leqslant 0.001$; **** : $P \leqslant 0.0001$.)
